# Clathrin Heavy Chain Knockdown Impacts CXCR4 Signaling and Post-translational Modification

**DOI:** 10.3389/fcell.2019.00077

**Published:** 2019-05-08

**Authors:** Maxwell S. DeNies, Luciana K. Rosselli-Murai, Santiago Schnell, Allen P. Liu

**Affiliations:** ^1^Cellular and Molecular Biology Graduate Program, University of Michigan Medical School, Ann Arbor, MI, United States; ^2^Department of Pharmacology, University of Michigan Medical School, Ann Arbor, MI, United States; ^3^Department of Molecular and Integrative Physiology, University of Michigan Medical School, Ann Arbor, MI, United States; ^4^Department of Computational Medicine and Bioinformatics, University of Michigan Medical School, Ann Arbor, MI, United States; ^5^Department of Mechanical Engineering, University of Michigan, Ann Arbor, MI, United States; ^6^Department of Biomedical Engineering, University of Michigan, Ann Arbor, MI, United States; ^7^Department of Biophysics, University of Michigan, Ann Arbor, MI, United States

**Keywords:** CXCR4, ERK signaling, membrane trafficking, clathrin, clathrin-mediated endocytosis, G proteincoupled receptor

## Abstract

Recent research has implicated endocytic pathways as important regulators of receptor signaling. However, the role of endocytosis in regulating chemokine CXC receptor 4 (CXCR4) signaling remains largely unknown. In the present work we systematically investigate the impact of clathrin knockdown on CXCR4 internalization, signaling, and receptor post-translational modification. Inhibition of clathrin-mediated endocytosis (CME) significantly reduced CXCR4 internalization. In contrast to other receptors, clathrin knockdown increased CXCL12-dependent ERK1/2 signaling. Simultaneous inhibition of CME and lipid raft disruption abrogated this increase in ERK1/2 phosphorylation suggesting that endocytic pathway compensation can influence signaling outcomes. Interestingly, using an antibody sensitive to CXCR4 post-translational modification, we also found that our ability to detect CXCR4 was drastically reduced upon clathrin knockdown. We hypothesize that this effect was due to differences in receptor post-translational modification as total CXCR4 protein and mRNA levels were unchanged. Lastly, we show that clathrin knockdown reduced CXCL12-dependent cell migration irrespective of an observed increase in ERK1/2 phosphorylation. Altogether, this work supports a complex model by which modulation of endocytosis affects not only receptor signaling and internalization but also receptor post-translational modification.

## Introduction

Tight regulation of how cells interact with their environment and process extracellular information is essential for survival. Consequentially, mammalian cells have evolved several tightly regulated yet partially redundant pathways to control cell communication (Scita and Di Fiore, 2010; McMahon and Boucrot, 2011; Liu et al., 2017). While endocytic pathways are predominantly known for their role in receptor internalization and desensitization, recent technological advancements have allowed researchers to interrogate endocytic dynamics in the context of cognate receptor-cargo pairs and systems-level studies have increased our understanding of endocytic pathways functioning a master regulators of receptor signaling, recycling, and degradation (Liu et al., 2009, 2010; Garay et al., 2015; English et al., 2018; Rosselli-Murai et al., 2018).

G protein-coupled receptors (GPCRs) play an important role for both essential and pathogenic biological processes and are a common cargo of endocytic pits. CXC chemokine receptor 4 (CXCR4) is a type 1 GPCR important for a variety of biological processes including immune cell homeostasis, embryonic development, and cell migration (Nagasawa et al., 1996; Ma et al., 1998; Zou et al., 1998; McGrath et al., 1999; Yi et al., 2014). CXCR4 signaling is activated by agonist C-X-C ligand 12 (CXCL12) binding (Busillo and Benovic, 2007). Depending on CXCL12 isoform and/or CXCR4 C-terminal tail post-translational modifications (PTM), CXCR4-CXCL12 signaling regulates cell proliferation and growth, chromatin remodeling, or cell migration and chemotaxis (Busillo and Benovic, 2007; Busillo et al., 2010; Ray et al., 2014; Yi et al., 2014). CXCR4 expression is deregulated in 23 cancers and has been shown to increase cancer cell metastasis toward CXCL12 expressing cells (Balkwill, 2004a,b). Aside from being the most commonly deregulated GPCR found in cancer, CXCR4 was also identified as an HIV co-receptor (Feng et al., 1996). Additionally, WHIM syndrome (Warts, Hypogammaglobulinemia, Infection, and Myelokathexis) is the only known immunological disease directly results from aberrant CXCR4 function, and it has been attributed to a deletion of C-terminal residues of CXCR4 that prevents proper receptor internalization (Diaz and Gulino, 2005).

Receptor tyrosine kinases and GPCRs such as CXCR4 commonly activate the ERK 1/2 signaling pathway in an agonist-dependent manner (Busillo et al., 2010; Lemmon and Schlessinger, 2010; Pinilla-Macua et al., 2016). ERK1/2 are serine/threonine protein kinase members in the MAPK signaling cascade and are involved in regulating numerous biological processes including cell migration and chemotaxis (Lewis et al., 1998; Pearson et al., 2001; Yoon and Seger, 2006). While the precise mechanism by which CXCL12 activates the MAPK cascade is unclear, the non-visual arrestins (beta 1 and 2) and CXCR4 PTMs have been implicated as key regulators of this process (Marchese et al., 2003; Bhandari et al., 2009; Busillo et al., 2010). Furthermore, multiple lines of evidence suggest tight spatial and temporal control is necessary for proper ERK1/2 activation (Malik et al., 2012). While the importance of spatiotemporal control and compartmentalization of receptor signaling have recently been shown to play a significant role in regulating receptor signaling, the molecular mechanisms that regulate this process remain largely unknown.

Recently, endocytosis and receptor localization in membrane microdomains have been implicated as key regulators of agonist-induced receptor signaling (Malik et al., 2012; Garay et al., 2015; Stone and Veatch, 2015; Stone et al., 2017). It was shown that drug perturbation as well as genetic silencing of clathrin heavy chain (CHC) significantly decreased EGF-induced EGFR internalization and AKT signaling and that these effects are rescued by ERBB2 expression in HeLa cells (Garay et al., 2015). Interestingly, EGFR surface expression and phosphorylation state was unaffected by clathrin inhibition (Garay et al., 2015). Additionally, recent work from our group suggested that a specific subset of clathrin-coated pits are specialized for signaling and concluded that clathrin acts as a scaffold for EGF-induced AKT phosphorylation at the cell membrane (Rosselli-Murai et al., 2018). Clathrin-independent endocytosis and lipid rafts have also been implicated as essential regulators of receptor signaling (Sigismund et al., 2008; Malik et al., 2012; Stone et al., 2017). In particular, disruption of caveolae by depleting cholesterol or caveolin-1 genetic silencing significantly reduced CXCL12-induced ERK1/2 signaling (Malik et al., 2012). Interestingly, this effect appeared to be CXCR4-specific as EGFR activation was unaffected by cholesterol depletion (Malik et al., 2012). Altogether, a model emerges in which endocytosis plays an essential role in ensuring complete receptor signaling pathway activation. While it is known that caveolin-1 plays an important role in CXCR4 signaling and CXCR4 is believed to primarily internalize *via* CME, it remains unclear how CME regulates CXCR4 signaling, PTMs, and protein levels.

In this study, we investigated the effects of clathrin genetic silencing on CXCR4 internalization, signaling, receptor protein levels, and PTM. We found that CHC knockdown significantly decreased CXCL12-induced CXCR4 internalization. In contrast to decreased ERK1/2 activation upon caveolin-1 knockdown, we observed an increased in ERK1/2 activation upon CHC knockdown. Using an antibody sensitive to CXCR4 PTM, we observed that increased signaling potential coincided with an increase in CXCR4 PTM, while total CXCR4 protein and mRNA levels were unaffected by clathrin knockdown. Interestingly, we also discovered that clathrin knockdown significantly impaired CXCL12-dependent cell migration irrespective of the observed increase in ERK1/2 phosphorylation. Altogether, our data support a more complex model in which clathrin is an important regulator of receptor signaling, internalization, and PTM.

## Results

### CXCR4 Overexpression in Retinal Pigment Epithelial (RPE) Cells Recapitulates Endogenous CXCR4 Internalization and Signaling in HeLa Cells

**Figure 1:**
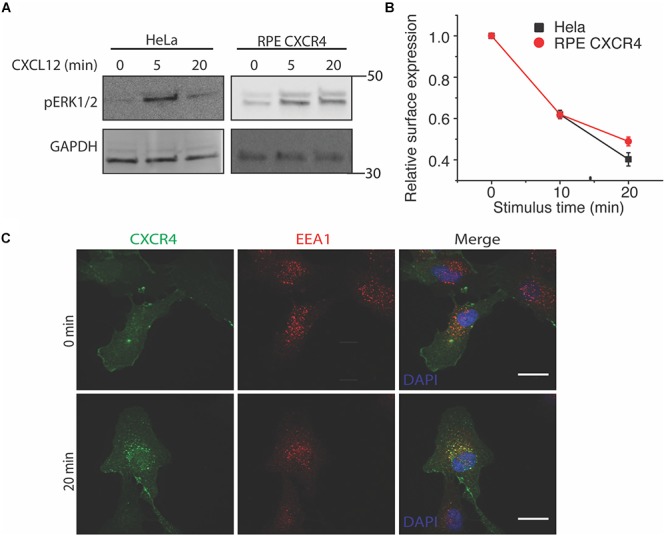
Overexpressed CXCR4 in RPE cells recapitulate endogenous CXCR4 signaling and internalization dynamics. **(A)** Representative western blot of CXCL12-induced ERK1/2 phosphorylation in HeLa and RPE cells overexpressing CXCR4. **(B)** Flow cytometry analysis of CXCR4 internalization in HeLa (endogenous) and RPE CXCR4 (overexpressed receptor). Relative surface expression was calculated by taking the mean fluorescence between a paired stimulus and vehicle control at each timepoint. The mean of 4 independent experiments is plotted ± SEM. **(C)** Confocal microscopy images of CXCR4 internalization labeled by FLAG antibody in RPE cells and an endosomal marker EEA1 antibody before and after 25 nM of CXCL12 treatment. Scale bars are 10 μm.

To study the effects of CXCR4 overexpression without background from endogenous receptors and other chemokine receptors responsive to CXCL12 (e.g., CXCR7), we used both HeLa and an exogenous CXCR4 overexpression cell line model. To limit background from endogenous CXCR4 and CXCR7, we overexpressed CXCR4 in retinal pigment epithelial cells (RPE) because this cell line has very low CXCR4 expression (Steel et al., 2014). As expected, CXCL12 stimulus rapidly induced both ERK1/2 phosphorylation in both HeLa and RPE cells stably overexpressing CXCR4 as early as the 5 min time point (Figure 1A). Likewise, agonist-induced receptor internalization was not significantly different between HeLa and RPE CXCR4 (Figure 1B). Lastly, to ensure that the overexpressed CXCR4 construct localized and trafficked properly in RPE cells, we found that CXCR4 colocalized with known early endosome marker EEA1 20 min post CXCL12 addition, as expected (Figure 1C).

### Clathrin Silencing Decreases CXCR4 Internalization and Increases CXCL12-Induced ERK1/2 Phosphorylation

**Figure 2:**
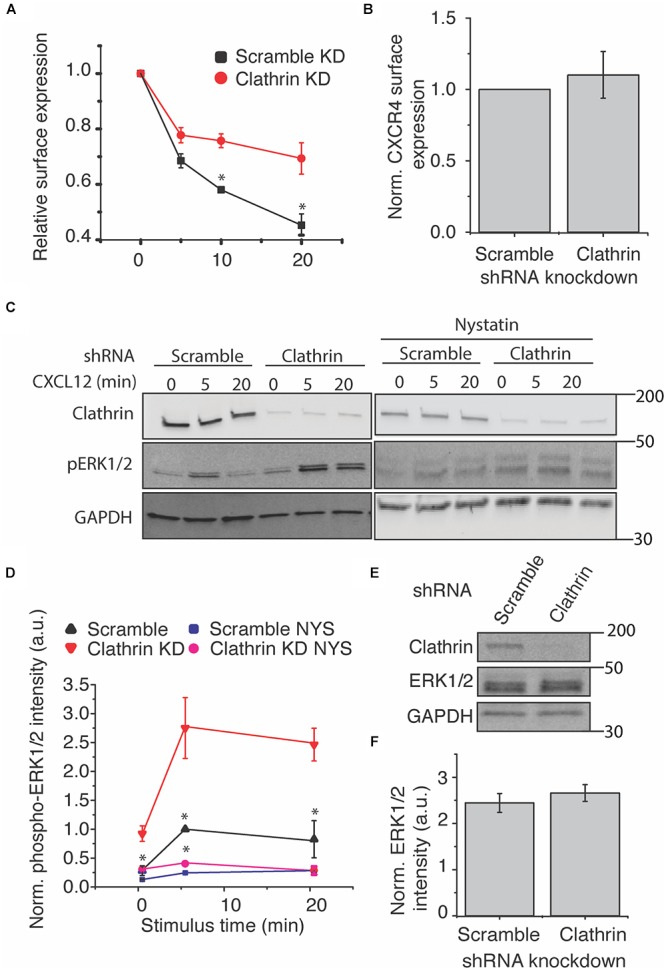
Clathrin inhibition reduces agonist-induced CXCR4 internalization and increases ERK1/2 phosphorylation. **(A)** Flow cytometry analysis of CXCL12-induced CXCR4 internalization upon scramble or clathrin knockdown (shRNA 2755 or 7981). Relative surface expression was calculated by taking the mean fluorescence between a paired stimulus and vehicle control at each time. The mean of 4 independent experiments is plotted ± SEM. **(B)** Quantification of relative CXCR4 surface expression upon clathrin knockdown (shRNA 2755 or 7981). CXCR4 surface expression was detected using the FLAG antibody in RPE cells overexpressing CXCR4 and measured by flow cytometry (*n* = 4, normalized mean ± SEM). **(C,D)** Representative western blots and quantification of CXCL12-induced ERK1/2 phosphorylation upon scramble or clathrin knockdown ± nystatin (NYS) treatment (50 μM, 30 min pretreatment). For all experiments, RPE cells overexpressing CXCR4 were transduced with either scramble or clathrin heavy chain shRNA (shRNA 2755) and stimulated with 25 nM CXCL12 for the labeled time course. Signaling experiments were conducted in pairs. Western blot bands were normalized to GAPDH as well as to 5 min stimulus in the scramble condition. The mean of 4 independent experiments is plotted ± SEM. **(E,F)** Representative western blot and quantification showing that relative ERK1/2 levels are unchanged upon clathrin (shRNA 2755) knockdown. Total ERK1/2 expression was normalized to GAPDH. The mean of 4 independent experiments is plotted ± SEM. (^∗^ denotes statistical significance *p* < 0.05).

Having established an experimental model to study CXCR4 in RPE cells, we next examined the effect of CHC knockdown on CXCR4 internalization and signaling. It has previously been hypothesized that CXCR4 is primarily internalized by CME (Dar et al., 2005). To test this hypothesis, we used shRNA to reduce functional clathrin triskelia and measured CXCL12-induced receptor internalization by flow cytometry. Consistent with this hypothesis, CXCR4 internalization was significantly attenuated, both in rate and in final level (Figure 2A). However, CXCR4 surface expression was unchanged by clathrin knockdown (Figure 2B). Next we investigated the effect of clathrin knockdown on CXCL12-CXCR4-mediated ERK1/2 signaling. In accordance with previous literature, we hypothesized that clathrin knockdown would reduce CXCL12-induced signaling. Surprisingly, clathrin knockdown significantly increased CXCL12-induced ERK1/2 phosphorylation both pre- and post-agonist addition (Figure 2C,D). To establish that these effects were not artifacts of receptor overexpression, we confirmed these observations in HeLa cells (Supplementary Figure 1). Previous reports have indicated that caveolae/lipid rafts are essential for complete CXCL12-mediated ERK1/2 phosphorylation (Malik et al., 2012). Consistent with this result, we found that inhibition of caveolae/lipid raft (using cholesterol depleting agent and caveolae inhibitor nystatin) reduced CXCL12-mediated ERK1/2 phosphorylation irrespective of clathrin knockdown (Figure 2C,D). As expected, total ERK1/2 levels were unchanged upon clathrin knockdown (Figure 2E,F). It has previously been reported that upon receptor stimulus, CXCR4 colocalizes with both adapter protein 2 (AP2) and caveolin-1 (Malik et al., 2012). Therefore, we hypothesized that clathrin knockdown may increase agonist-induced CXCR4 colocalization with caveolin-1. While not statistically significant, a slight increase in CXCR4 colocalization with caveolin-1 was observed upon clathrin knockdown after 5 min agonist addition (Supplementary Figures 2A,B). In contrast to previous work, CXCR4 colocalization with caveolin-1 was limited irrespective of clathrin knockdown. However, others have shown that CXCR4 localizatizes to lipid rafts more generally (Nguyen et al., 2005; Chinni et al., 2008). Thus, it is plausible that clathrin knockdown may shift CXCR4 localization to both caveolin-1 positive and negative lipid rafts. Together, these observations support a model where endocytic pathway redundancy may play a role in modulating receptor internalization and signaling. Interestingly, a statistically significant increase in ERK1/2 phosphorylation was also observed at the 0 min time point (Figure 2D) in RPE cells overexpressing CXCR4. We believe that this might be due to ineffective clearance of activated CXCR4 during serum starvation as receptor internalization is significantly reduced upon clathrin knockdown (Figure 2A). An increase in ERK1/2 phosphorylation at the 0 min time point was not observed with HeLa cells with clathrin knockdown. We believe that this discrepancy is likely due to cell type specificity or difference in signal intensity and consequently detection sensitivity.

### The CXCR4 UMB2 Antibody Immunoblotting Is a Robust Method to Detect Changes in CXCR4 PTM

**Figure 3:**
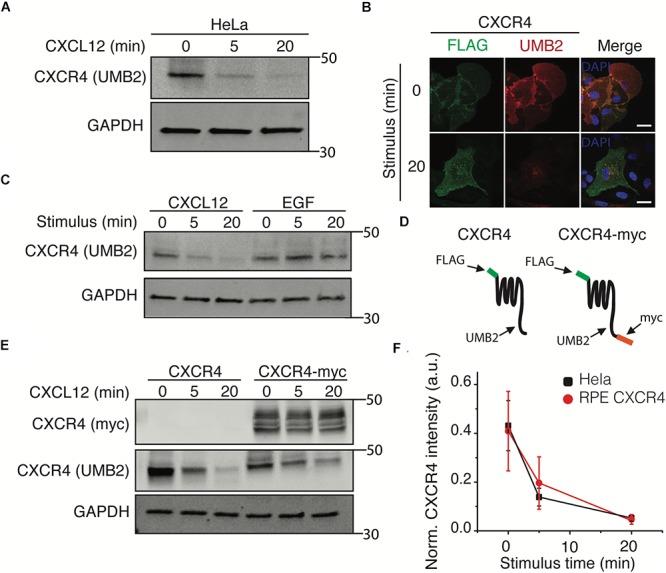
The UMB2 monoclonal CXCR4 antibody detects changes in agonist-induced CXCR4 PTMs. **(A)** Representative western blot of CXCR4 detection upon CXCL12 addition in HeLa cells. Cells were stimulated with 25 nM for the described time course and CXCR4 protein levels were quantified using the UMB2 monoclonal antibody and normalized to GAPDH. **(B)** Confocal microscopy images of overexpressed FLAG-tagged CXCR4 in RPE cells. CXCR4 was labeled using both FLAG and UMB2 antibodies pre- and post-CXCL12 addition (25 nM). Scale bars are 10 μm). **(C)** Reduced detection of CXCR4 using the UMB2 antibody is ligand-specific. RPE cells overexpressing CXCR4 were treated with either 25 nM CXCL12 or 20 nM EGF for the described time course. **(D)** Schematic of CXCR4 and CXCR4-myc overexpression constructs illustrating where the different antibodies used bind to CXCR4. **(E)** RPE cells overexpressing CXCR4-myc were treated with 25 nM CXCL12 or 20 nM EGF for the described time course and CXCR4 was detected by western blotting using the myc or UMB2 antibody. **(F)** Western blot quantification of CXCR4 detection by UMB2 antibody in HeLa and RPE CXCR4 cells. CXCR4 intensity was normalized to GAPDH and plotted mean ± SEM from 4 independent experiments.

To further investigate the mechanism by which clathrin knockdown increased CXCR4 signaling, we tested whether clathrin knockdown influenced CXCR4 protein expression. Upon conducting these experiments, we noticed that the CXCR4 UMB2 antibody differentially detected CXCR4 post-receptor stimulus (Figure 3A). To validate that the decrease in receptor western blot intensity was not due to rapid receptor degradation, we co-labeled overexpressed FLAG-CXCR4 with FLAG and UMB2 antibodies pre- and post-CXCL12 stimulus. Consistent with our hypothesis, the FLAG antibody robustly detected CXCR4 pre- and post-CXCL12 stimulus while the UMB2 antibody signal was significantly reduced post-stimulus (Figure 3B). To confirm that the UMB2 disappearance was dependent on CXCL12, we stimulated cells with EGF and monitored relative CXCR4 levels. As expected, while CXCL12 addition quickly led to the disappearance of the UMB2 CXCR4 band, EGF stimulus did not affect CXCR4 detection (Figure 3C). To exclude that CXCR4 was moving to an insoluble compartment post-stimulus, we generated a stable cell line expressing a c-terminally tagged myc CXCR4 construct (CXCR4-myc) (Figure 3D). As expected, while the CXCR4 UMB2 antibody’s ability to detect CXCR4 post-stimulus was diminished, the myc antibody staining remained constant (Figure 3E), and hence we do not believe the receptors were degraded. Since the cytosolic c-terminal tail of CXCR4 is rapidly post-translationally modified by phosphorylation and ubiquitination upon CXCL12 addition, our results suggest that the UMB2 antibody is sensitive to changes in CXCR4 PTM. Equipped with this new tool, we asked whether overexpressed CXCR4 had different PTM kinetics than endogenous CXCR4, and observed no significant changes in CXCR4 PTM kinetics between HeLa and RPE CXCR4 cells (Figure 3F).

### Clathrin Inhibition Increases CXCR4 PTMs

**Figure 4:**
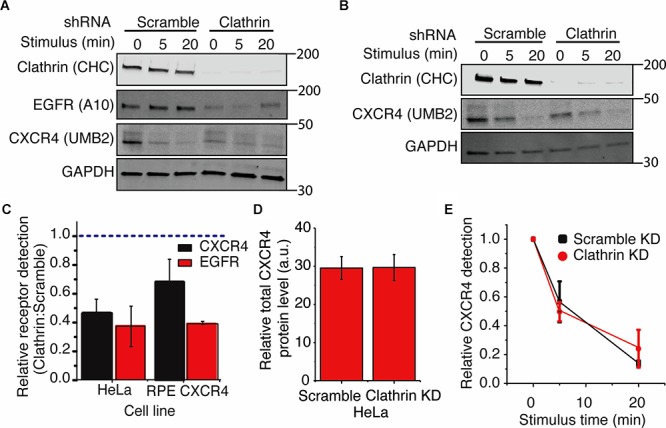
Clathrin knockdown increases CXCR4 PTM. **(A,B**) Representative western blots showing the effect of clathrin knockdown on CXCR4 and EGFR protein levels in **(A)** HeLa or **(B)** RPE CXCR4 cells. **(C)** Quantification of western blot analysis of CXCR4 and EGFR protein levels upon clathrin knockdown. Relative receptor detection was calculated by taking the ratio of normalized receptor intensity (receptor:GAPDH) between scramble and clathrin shRNA transduced cells (0 min time point). Cells were transduced with scramble or clathrin shRNA (2755) for 5 days and serum-starved for 4 hrs prior to each signaling/receptor detection experiment. Quantification is mean ± SEM from 4 independent experiments. **(E)** Relative detection of CXCR4 by UMB2 with and without clathrin knockdown in RPE cells overexpressing CXCR4. Relative CXCR4 detection was calculated upon receptor stimulus (25 nM CXCL12) for scramble and clathrin knockdown cells. CXCR4 detection was normalized to the 0 min time point and plotted as mean ± SEM (*n* = 4). Similar analysis was not carried out for HeLa cells as the signal was outside of the linear range of detection (see **A**). **(D)** Total endogenous CXCR4 detection in HeLa cells. Cells were infected with either clathrin or scramble shRNA and serum-starved for 4 h. After permeabilization and immunofluorescence labeling of endogenous CXCR4, total CXCR4 protein level was measured by flow cytometry. Mean ± SEM is plotted from 3 independent experiments.

Using the UMB2 antibody, we observed that clathrin knockdown significantly reduced CXCR4 detection in both HeLa and RPE cells overexpressing CXCR4 (Figure 4A–C). To determine whether this was due to a change in receptor expression or PTM state, we used flow cytometry and qPCR to assess total endogenous CXCR4 protein and mRNA levels upon clathrin knockdown. Interestingly, clathrin inhibition did not affect total endogenous CXCR4 protein (Figure 4D) or mRNA levels in HeLa cells (Supplementary Figures 3A,B). Cycloheximide chase experiments in RPE cells overexpressing c-terminal myc tagged CXCR4 further confirmed that clathrin knockdown did not lead to a change in CXCR4 degradation kinetics (Supplementary Figures 3C,D) and additionally total CXCR4 labeling using the myc antibody confirmed that CXCR4 total protein levels were unchanged in the overexpression CXCR4 model as well (Supplementary Figure 3E). To test whether this phenomenon was specific to CXCR4 or more broadly applicable to other receptors, we measured EGFR protein levels using an antibody (A-10 clone) not expected to be sensitive to receptor PTM (Wee and Wang, 2018). Interestingly, clathrin knockdown significantly reduced EGFR detection in both HeLa and RPE cells as well. EGFR mRNA levels were also unchanged (Supplementary Figures 3C,D). Since EGFR is internalized by both CME and clathrin-independent internalization (Sigismund et al., 2005, 2008, 2013), it is possible that upon clathrin knockdown EGFR internalization is compensated by clathrin-independent internalization and consequentially leads to increased receptor degradation as previously reported. Reliable quantification of changes of CXCR4 PTM kinetics was not possible for the clathrin knockdown condition in HeLa cells due to the limited linear detection range of fluorescent imagers since these cells had a drastically lower initial CXCR4 detection intensity. Consequentially, we explored whether clathrin knockdown had any effect on CXCR4 PTM kinetics in the overexpression context. As previously reported with EGFR (Garay et al., 2015), we did not see a significant change in agonist-induced CXCR4 PTM kinetics upon clathrin knockdown in RPE cells overexpressing CXCR4 (Figure 4E).

### Clathrin Knockdown Decreases CXCL12-Dependent and Independent Cell Migration in HeLa Cells

**Figure 5:**
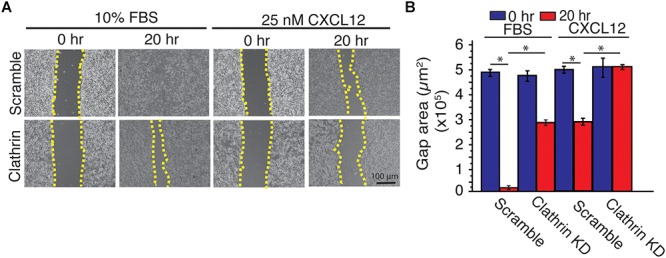
Clathrin knockdown decreased CXCL12-dependent and independent cell migration in HeLa cells. **(A)** Representative images of a scratch assay time course. HeLa cells transduced with scramble or clathrin shRNA (shRNA 2755 or 7981) (day 5) were serum-starved for 4 h and treated with medium containing 10% FBS or 25 nM CXCL12 in serum-free medium. A line was scratched on each plate and relative cell migration was measured by phase contrast microscopy at 0 or 20 h post-scratching. Cell boundaries used to calculate cell migration are outlined in yellow dotted line. **(B)** Quantification of gap area (between dotted yellow boundaries) under each condition. Mean gap area ± SD is plotted from 4 independent experiments (^∗^ denotes statistical significance *p* < 0.05).

To investigate the implication of CXCL12-induced increase of ERK1/2 phosphorylation in clathrin-silenced cell on cell migration, we used a wound-healing assay. Compared to scramble controls, irrespective of the type of ligand (CXCL12 vs. FBS), clathrin knockdown significantly reduced cell migration in HeLa cells (Figure 5). This result corroborated a previous report where compared to a wildtype receptor, a mutant CXCR4 has indistinguishable ERK1/2 phosphorylation yet has significantly reduced chemotaxis (Mines et al., 2009). Since EGF- and CXCL12-mediated ERK1/2 phosphorylation have been shown to occur at distinct membrane micro-domains (Malik et al., 2012), we investigated how EGF-dependent cell migration was affected by clathrin knockdown. Similar to CXCL12, clathrin knockdown reduced EGF-induced cell migration (Supplementary Figures 4A,B). This suggests that while CXCL12 and EGF signaling mechanisms are distinct and differentially regulated by clathrin, the effects on cell migration are comparable.

## Discussion

The expanded role of endocytosis in not only regulating receptor internalization but as a signaling platform and master regulator of protein expression is becoming increasingly clear (Sorkin and von Zastrow, 2009). Here we provide new evidence of how endocytic proteins regulate CXCR4 internalization, signaling, and receptor PTM level and highlight the redundancy of endocytic pathways.

Redundancy of endocytic pathways is not a new idea and work in this area has been pioneered with EGFR (Sigismund et al., 2005). Interestingly, we found that while clathrin knockdown increased CXCR4 PTM, this was not the case for EGFR as the antibody used to measure total EGFR has been previously shown to not be sensitive to EGFR PTMs (Wee and Wang, 2018) and previous research suggests that EGFR PTMs are unaffected by clathrin inhibition (Garay et al., 2015). Additionally, while EGFR is preferentially internalized by CME at increasing high ligand levels, at times it is also internalized by clathrin-independent mechanisms (Sigismund et al., 2005). It was further reported that the endocytic mechanism of EGFR internalization predestined receptors for either receptor recycling or degradation in a PTM specific manner (Sigismund et al., 2005, 2008, 2013). Consequently, it is possible that clathin knockdown leads to an increase in clathrin-independent EGFR internalization and subsequent degradation and provides additional evidence that clathrin inhibition differentially impacts receptors such as EGFR and CXCR4.

Upon clathrin knockdown, we observed an increase in CXCR4 PTM pre-stimulus. However, total as well as surface receptor levels remained unchanged. Since clathrin is involved in multiple aspects of endocytic trafficking (Golgi transport as well as endocytosis), it is possible that clathrin knockdown may lead to changes in CXCR4 localization in plasma membrane micro-domains or intracellular compartments that are responsible for the observed change in receptor PTM. Even though CXCR4 is both phosphorylated and ubiquitinated, we did not discern which modification or combination of modifications were responsible for these observations and this remains to be a subject of further investigation.

Interestingly, increased CXCR4 PTM correlated with CXCL12-dependent ERK1/2 phosphorylation, further implicating compensatory mechanisms of CXCR4 regulation upon clathrin knockdown. It was previously reported that CXCL12-induced ERK1/2 phosphorylation occurs independently of CXCR4 internalization and that CXCR4 localization in cholesterol rich lipid rafts is essential for complete ERK1/2 phosphorylation and that this effect was not observed with EGFR receptor (Malik et al., 2012). Indeed, transient silencing of the caveolae resident protein caveolin-1 was reported to lead to an approximately 50% reduction in CXCL12-induced ERK1/2 phosphorylation (Malik et al., 2012). Together, these results provide additional evidence that modulation of endocytic dynamics regulates receptor biology differently. For CXCR4, clathrin appears to be a negative regulator of ERK1/2 phosphorylation and increased receptor PTMs, whereas for EGFR ERK1/2 signaling is unaffected and total protein levels are reduced during extended periods of clathrin knockdown.

Interestingly, beta-arrestin (1 and 2) knockdown phenocopies ERK1/2 phosphorylation that we observed with clathrin knockdown (Malik et al., 2012). Together these results suggest a potential shared mechanism by which clathrin and beta-arrestins are negative regulators of CXCR4-dependent ERK1/2 signaling and a compensatory mechanism by which cells increase CXCR4 localization in lipid rafts (caveolin-1 positive and/or negative) and become increasingly capable for CXCL12-induced ERK1/2 phosphorylation. Additionally, while AP2 inhibition has been shown to decrease CXCR4 internalization, it has not been shown to affect ERK1/2 signaling (Malik et al., 2012). Coupled with our results, this provides evidence for a model where AP2 is important for CXCR4 signaling but an independent adapter protein is important for mediating CXCL12-dependent ERK1/2 phosphorylation. Unfortunately, co-knockdown experiments of clathrin and caveolin-1 resulted in cells that were not viable. However, consistent with the model, it is possible that clathrin knockdown increases clathrin-independent internalization of CXCR4 (clathrin knockdown slightly increased CXCR4/caveolin-1 colocalization) and that unlike EGFR, this mechanism of internalization does not lead to increased receptor degradation.

Surprisingly, a significant increase in ERK1/2 phosphorylation was observed at the 0 min time point in CXCR4 RPE cells upon clathrin knockdown. We speculate that this observation could be due to the inability of clathrin knockdown cells to efficiently clear activated receptor during serum starvation. This is supported by the observation that clathrin inhibition drastically reduced CXCR4 internalization. Interestingly, elevated ERK1/2 phosphorylation did not lead to increased cell migration. Clathrin silencing nearly ablated cell migration independent of agonist and our observation that clathrin knockdown increases ERK1/2 signaling suggests that CXCL12-induced ERK1/2 signaling and cell migration are decoupled or that there may be additional roles that clathrin plays in cell migration. While clathrin inhibition has not been previously associated with a change in ERK1/2 signaling, previous studies with CXCR4 have focused on transient pharmacological inhibition of CME (30 min timescale) (English et al., 2018), whereas in this study shRNAs were used to specifically knockdown CHC over the course of 3–5 days) and consequently provides additional evidence for cellular compensation. Additionally, this effect appears to be different in HL-60 derived neutrophils where inhibition of clathrin abrogated ERK1/2 signaling and polarization (Tan et al., 2018). This could be due to a strong dependence of signaling on the clathrin scaffold for a professional migratory cell as well as differences between pharmacological vs. genetic perturbations. Our present findings are supported by the work of Mines et al. (2009) that used a lysine triple mutant CXCR4. They showed that this mutant CXCR4 had significantly reduced chemotactic potential independent of ERK1/2 phosphorylation. Together with the results presented, this evidence supports a model in which CXCL12-induced chemotaxis and cell migration is decoupled from CXCL12-induced ERK1/2 phosphorylation. We additionally investigated how EGF-dependent cell migration was affected upon clathrin knockdown. Irrespective of ligand, clathrin knockdown significantly reduced cell migration. This suggests that while CXCL12 and EGF signaling mechanisms are distinct (different membrane localization) and differentially regulated by clathrin, the effects on cell migration are similar. Additionally, it is likely that clathrin is impacting cell migration *via* an ERK1/2-independent mechanism.

In addition to advancing our knowledge of how endocytosis modulates CXCR4 biology, we also report that the UMB2 antibody differentially detects CXCR4 post-receptor stimulus. This effect is agonist specific, independent of receptor internalization, and not due to differential protein extraction. While not directly tested in this work, and since the UMB2 antibody binds to the c-terminus of the receptor, we believe that the observed effect is due to changes in CXCR4 PTM. As CXCR4 is rapidly post-translationally modified upon receptor-ligand binding, it is plausible that these modifications interfere with antibody binding. In agreement with previous work with EGFR, our results support that clathrin silencing does not modulate receptor PTM kinetics (Garay et al., 2015).

While our study along with a growing number of others supports a dynamic model of compensatory endocytic mechanism working together to precisely regulate receptor biology, additional research is necessary to determine whether these observations are direct effects of endocytic adapter protein-GPCR interactions or secondary effects of modulating this essential cellular process. Additionally, due to the extraordinary cell type and tissue specificity of GPCR expression, it is necessary to interpret these experiments within this context and it will be important to determine whether these observations are tissue- and/or GPCR-specific or more broadly applicable to other receptors. Together with knowledge that endocytic dynamics are modulated by cancer (Schmid, 2017; Rosselli-Murai et al., 2018), further mechanistic investigations of how CME and clathrin-independent endocytosis regulate receptor biology is likely to reveal novel signaling mechanism that may provide new therapeutic strategies to selectively target pathogenic GPCR signaling such as CXCR4 in metastatic cancers.

## Experimental Materials and Methods

### Cell Culture

HeLa Cells were originally obtained from ATCC. RPE cells were obtained from Dr. Sandra Schmid at UT Southwestern and all stable cell lines were derived directly from this line. HeLa cells were cultured in DMEM media (Corning) supplemented with 10% FBS while RPE cell lines were cultured in DMEM/F12 media (Corning) supplemented with 10% FBS.

### Stable Cell Lines

Stable RPE cell lines expressing FLAG-CXCR4 WT (± c-terminal myc tag) receptors were generated using lentiviral transduction produced from the pLVX vector. Lentiviruses were generated in our lab by co-transfecting HEK293T cells (ATCC) with the transfer plasmid, psPAX2, and pMD2.G lentiviral envelope and packaging plasmids. Cell supernatant containing mature lentiviral particles was collected 4 days after transfection. RPE cells were transduced in DMEM/F12 media supplemented with FBS and 10 μg/mL polybrene and stable cell lines were generated through puromycin selection (3 μg/mL).

### Cell Signaling, Cycloheximide Experiments, and Western Blotting

Cells were plated onto 12 well plates (Cell Treat) 24 h before each signaling experiment. Prior to each experiment cells were wash 1× with PBS and serum-starved for 4 h. Cells were stimulated with ligand (specified in figure legends) (R&D Systems) diluted in serum-free media for 0, 5, or 20 min as indicated in each figure legend. For clathrin knockdown experiments, cells were transduced with pKLO.1 clathrin heavy chain (CHC 17) (sequence 1: Sigma TRCN0000342755, CCGGCGGTTGCTCTTGTTACGGATACTCGAGTATCCGTAACAAGAGCAACCGTTTTTG (2755), sequence 2: Sigma TRCN0000007981, CCGGCGTGTTCTTGTAACCTTTATTCTCGAGAATAAAGGTTACAAGAACACGTTTTT (7981; Supplementary Figure 5) or scramble non-targeting control Sigma-SCH002) shRNA containing lentiviral particles 5 days prior to the signaling experiment. Seventy two hours post-transduction, cells were selected using 2 μg/mL (HeLa) or 3 μg/mL puromycin (RPE). Knockdown efficiencies ranged from 55 to 85% for all experiments. Cells were plated 24 h prior to each signaling experiment as described above. After each signaling experiment, cells were washed with PBS 1× and lysed using RIPA buffer (Pierce) supplemented with protease (EDTA Peirce Cocktail and phosphatase inhibitor cocktails (HALT Phosphatase Inhibitor) for 10 min on ice. Afterward, cells were scraped, lysate added to 1.5 mL tubes, and centrifuged at 4°C for 45 min at 16,100 g. Approximately 10–50 μg of protein were loaded for SDS-PAGE gels (BioRad) analysis depending on the abundance of the target protein. PDVF 0.2 μm membranes (Thermo Fisher Scientific) were used for all western blotting experiments and transferred using the iBlot transfer system suitable for mixed range proteins. Blots were blocked using 5% BSA in TBS not supplemented with Tween for 1 hr rocking at room temperature. Blots were then incubated overnight with primary antibodies diluted in 5% BSA supplemented with 1% Tween 20 in TBS (TBST). Blots were washed 3× for 5 min with TBST after which they were incubated with secondary antibodies (Table 1). All blots were imaged using the LiCor Odyssey Imaging System. Bands were quantified using the LiCor Image Analysis Software in accordance with manufacturer guidelines. We established that our knockdowns did not significantly alter GAPDH expression using the REVERT total Protein Stain and therefore used GAPDH as a loading control and for normalization of all experiments. As previously described, all signaling experiments were additionally normalized to the scramble control at the 5 min stimulus time point (Garay et al., 2015). For the cycloheximide (CHX) experiments, RPE cells overexpressing CXCR4 were grown as described above and treated with cycloheximide (50 μM) described in the figure legend. The exact western blot protocol used for signaling assays was used for CHX experiments as well. All statistics were conducted using paired 2 tailed *t*-test (^∗^ represents *p* < 0.05).

**Table 1:** Reagents

Reagent	Supplier	Dilution	Assay
ms-FLAG-647	Genscript	1:1,000	FC, IF
Gt-anti-rb Dylight 800	Invitrogen	1:10,000	WB
Gt-anti-ms Dylight 680	Invitrogen	1:10,000	WB
Human CXCR4 R-PE	Invitrogen	1:500	FC
ms-GAPDH	Santa Cruz	1:1,000	WB
rb-Phospho-pERK1/2	CST	1:2,000	WB
ms-Total ERK1/2	CST	1:1,000	WB
rb-CXCR4 UMB2	Abcam	1:1,000	WB, IF
Rb-EEA1	CST	1:500	IF
Gt-anti-rb-AF488	Invitrogen	1:1,000	IF
Rb-Caveolin-1	CST	1:500	IF
Ms-EGFR (A-10)	Santa Cruz	1:1,000	WB

### Immunofluorescence Assays

Cells were plated onto 6-well plates on glass coverslips 24 h prior to each experiment. Cells were washed 1× with PBS and serum-starved for 4 h as described above. Cells were stimulated as specified in each figure legend and immediately washed with cold PBS 2× and fixed in 4% paraformaldehyde for 15 min on ice. Cells were permeabilized for 5 min at room temperature with 0.2% Triton-X100 diluted in PBS and then blocked with 2.5% BSA in PBS for 1 h. Cells were incubated with primary antibody diluted in 2.5% BSA and incubated overnight at 4°C (Table 1). Slides were wash 3× for 5 min each with PBS and incubated with secondary antibodies diluted in 2.5% BSA for 1 h at room temperature (Table 1). Cells were washed with PBS 3×, 5 min per wash and incubated with DAPI (Table 1) diluted in PBS for 5 min at room temperature. Afterward, cells were washed with PBS and mounted onto glass slides with Fluoromount-G (Invitrogen). Slides were imaged by spinning disk confocal microscopy, TIRF microscopy, or epifluorescence (using a laser-based system). Different experimental samples were imaged using the same imaging settings each day and antibody controls (secondary and non-permeabilized samples) were included in each experiment to account for background staining.

### Internalization Assays

Cells were plated onto 10 cm dishes 24 h prior to each experiment, washed and serum-starved as described above. Afterward, cells were non-enzymatically disassociated from each plate using 50 μM EDTA in Ca^2+^-free PBS. Cells were pelleted by centrifugation (500 g for 10 min) and resuspended in serum-free media on ice. Cells were transferred into 1.5 mL eppendorf tube and treated with either a vehicle or ligand and transferred to a hot plate at 37°C for a time course (described in figure legends). Immediately afterward, cells were transferred back on ice, pelleted (centrifuged 3,000 g for 3 min), and resuspended in 4% paraformaldehyde for 10 min on ice. Cells were pelleted and incubated with conjugated antibody (Table 1) diluted in 2.5% BSA for 1.5 h on ice. Afterward, cells were washed 1× with PBS and 10,000 cells were analyzed by the Guava EasyCyte Flow Cytometer and its accompanied software. Internalization was quantified as previously described (Marchese and Benovic, 2001). All statistics were conducted using paired 2 tailed *t*-test (^∗^ represents *p* < 0.05).

### RT-qPCR Assays

Scramble- or clathrin-silenced HeLa cells were plated onto 12 well dishes at similar confluency 24 h prior to each experiment and subsequently serum-starved or treated with fresh 10% FBS containing medium for 4 hr. RNA was extracted using the RNAeasy kit (BioRad) and 1 μg of cDNA prepared in accordance with the iScripts cDNA synthesis protocol (BioRad). qPCR assays were conducted using SYBR Green (BioRad) per BioRad protocol instructions. Primers used to quantify *gapdh* (Fw-GAGTCAACGGATTTGGTCGT, Rev-CTTGATTTTGGAGGGATCTCGC), *actin* (Fw-CATGTACGTTGCTATCCAGGC, Rev-CTCCTTAATGTCACGCACGAT), *chc* (Fw-ACAGAGACACAACCCATTGTTT, Rev-CGGTGGTGCGGTATAACCAT), *cxcr4* (Fw-CCTATGCAAGGCAGTCCATGT, Rev-GGTAGCGGTCCAGACTGATGA), and *egfr* (Fw-AGGCACGAGTAACAAGCTCAC, Rev-ATGAGGACATAACCAGCCACC). Relative transcript levels were quantified using the ΔΔC_t_ method as previously described. Briefly, the change in C(t) values between scramble and clathrin knockdown samples were determined. Afterward the change between the deltas of paired scramble and clathrin knockdowns was calculated (i.e., ΔΔC_t_) and relative transcript levels was determined by calculating 2^-ΔΔCt^. Results were collected in duplicate from 4 independent experiments; log_2_ transformed, and plotted mean ± standard deviation.

### Migration Assays

HeLa cells expressing either scramble or CHC shRNA were plated onto 24 well plates at 100% confluence. After 24 h, cells were serum-starved (DMEM (-) FBS) for 4 hr and a vertical scratch was created using a 200 μL pipet tip. Afterward, cells were treated with either complete media (10% FBS + DMEM) or CXCL12 media (25 nM CXCL12 + DMEM). Cell migration was monitored, imaged and quantified after 20 h using phase contrast microscopy on a Cytation 5 automated plate reader at 4× magnification. Migration was quantified by measuring the area remaining between the cell borders by using ImageJ as previously described (Rosselli-Murai et al., 2018). Results were collected from 3 independent experiments and are plotted mean ± standard deviation. All statistics were conducted using paired 2 tailed *t*-test (^∗^ represents *p* < 0.05).

## Author Contributions

MD conceived, designed, carried out experiments, analyzed data, and wrote the manuscript. LR-M contributed to reagent generation, data acquisition, and data interpretation. SS contributed to data interpretation. AL conceived experiments and wrote the manuscript.

## Conflict of Interest Statement

The authors declare that the research was conducted in the absence of any commercial or financial relationships that could be construed as a potential conflict of interest.

## Abbreviations

CHC: Clathrin heavy chain
CME: Clathrin-mediated endocytosis
CXCR4: C-X-C Chemokine Receptor 4
EGFR: Epidermal growth factor receptor
PTMs: post-translational modifications.

## References

[B1] Balkwill F. (2004a). Cancer and the chemokine network. *Nat. Rev. Cancer* 4 540–550. 10.1038/nrc1388 15229479

[B2] Balkwill F. (2004b). The significance of cancer cell expression of the chemokine receptor CXCR4. *Semin. Cancer Biol.* 14 171–179. 10.1016/j.semcancer.2003.10.003 15246052

[B3] Bhandari D. Robia S. L. Marchese A. (2009). The E3 ubiquitin ligase atrophin interacting protein 4 binds directly to the chemokine receptor CXCR4 via a novel WW domain-mediated interaction. *Mol. Biol. Cell* 20 1324–1339. 10.1091/mbc.E08-03-0308 19116316 PMC2649280

[B4] Busillo J. M. Armando S. Sengupta R. Meucci O. Bouvier M. Benovic J. L. (2010). Site-specific phosphorylation of CXCR4 is dynamically regulated by multiple kinases and results in differential modulation of CXCR4 signaling. *J. Biol. Chem.* 285 7805–7817. 10.1074/jbc.M109.091173 20048153 PMC2844224

[B5] Busillo J. M. Benovic J. L. (2007). Regulation of CXCR4 signaling. *Biochim. Biophys. Acta* 1768 952–963. 10.1016/j.bbamem.2006.11.002 17169327 PMC1952230

[B6] Chinni S. R. Yamamoto H. Dong Z. Sabbota A. Bonfil R. D. Cher M. L. (2008). CXCL12/CXCR4 transactivates HER2 in lipid rafts of prostate cancer cells and promotes growth of metastatic deposits in bone. *Mol. Cancer Res.* 6 446–457. 10.1158/1541-7786.MCR-07-0117 18337451 PMC3842603

[B7] Dar A. Goichberg P. Shinder V. Kalinkovich A. Kollet O. Netzer N. (2005). Chemokine receptor CXCR4–dependent internalization and resecretion of functional chemokine SDF-1 by bone marrow endothelial and stromal cells. *Nat. Immunol.* 6 1038–1046. 10.1038/ni1251 16170318

[B8] Diaz G. A. Gulino A. V. (2005). WHIM syndrome: a defect in CXCR4 signaling. *Curr. Allergy Asthma Rep.* 5 350–355. 10.1007/s11882-005-0005-0 16091205

[B9] English E. J. Mahn S. A. Marchese A. (2018). Endocytosis is required for C-X-C chemokine receptor type 4 (CXCR4)-mediated Akt activation and anti-apoptotic signaling. *J. Biol. Chem.* 293 11470–11480. 10.1074/jbc.RA118.001872 29899118 PMC6065176

[B10] Feng Y. Broder C. C. Kennedy P. E. Berger E. A. (1996). HIV-1 entry cofactor: functional cDNA cloning of a seven-transmembrane, G protein-coupled receptor. *Science* 272 872–877. 10.1126/science.272.5263.872 8629022

[B11] Garay C. Judge G. Lucarelli S. Bautista S. Pandey R. Singh T. (2015). Epidermal growth factor–stimulated akt phosphorylation requires clathrin or ErbB2 but not receptor endocytosis. *Mol. Biol. Cell* 26 3504–3519. 10.1091/mbc.E14-09-1412 26246598 PMC4591694

[B12] Lemmon M. A. Schlessinger J. (2010). Cell signaling by receptor tyrosine kinases. *Cell* 141 1117–1134. 10.1016/j.cell.2010.06.011 20602996 PMC2914105

[B13] Lewis T. S. Shapiro P. S. Ahn N. G. (1998). Signal transduction through MAP kinase cascades. *Adv. Cancer Res.* 74 49–139. 10.1016/s0065-230x(08)60765-4 9561267

[B14] Liu A. P. Aguet F. Danuser G. Schmid S. L. (2010). Local clustering of transferrin receptors promotes clathrin-coated pit initiation. *J. Cell Biol.* 191 1381–1393. 10.1083/jcb.201008117 21187331 PMC3010081

[B15] Liu A. P. Botelho R. J. Antonescu C. N. (2017). The big and intricate dreams of little organelles: embracing complexity in the study of membrane traffic. *Traffic* 18 567–579. 10.1111/tra.12497 28574194

[B16] Liu A. P. Loerke D. Schmid S. L. Danuser G. (2009). Global and local regulation of clathrin-coated pit dynamics detected on patterned substrates. *Biophys. J.* 97 1038–1047. 10.1016/j.bpj.2009.06.003 19686651 PMC2726330

[B17] Ma Q. Jones D. Borghesani P. R. Segal R. A. Nagasawa T. Kishimoto T. (1998). Impaired B-lymphopoiesis, myelopoiesis, and derailed cerebellar neuron migration in CXCR4- and SDF-1-deficient mice. *Proc. Natl. Acad. Sci. U.S.A.* 95 9448–9453. 10.1073/pnas.95.16.9448 9689100 PMC21358

[B18] Malik R. Soh U. J. K. Trejo J. Marchese A. (2012). Novel roles for the E3 ubiquitin ligase atrophin-interacting protein 4 and signal transduction adaptor molecule 1 in G protein-coupled receptor signaling. *J. Biol. Chem.* 287 9013–9027. 10.1074/jbc.M111.336792 22275353 PMC3308791

[B19] Marchese A. Benovic J. L. (2001). Agonist-promoted ubiquitination of the G protein-coupled receptor CXCR4 mediates lysosomal sorting. *J. Biol. Chem.* 276 45509–45512. 10.1074/jbc.C100527200 11641392

[B20] Marchese A. Raiborg C. Santini F. Keen J. H. Stenmark H. Benovic J. L. (2003). The E3 ubiquitin ligase AIP4 mediates ubiquitination and sorting of the G protein-coupled receptor CXCR4. *Dev. Cell* 5 709–722. 10.1016/S1534-5807(03)00321-6 14602072

[B21] McGrath K. E. Koniski A. D. Maltby K. M. McGann J. K. Palis J. (1999). Embryonic expression and function of the chemokine SDF-1 and its receptor. CXCR4. *Dev. Biol.* 213 442–456. 10.1006/dbio.1999.9405 10479460

[B22] McMahon H. T. Boucrot E. (2011). Molecular mechanism and physiological functions of clathrin-mediated endocytosis. *Nat. Rev. Mol. Cell Biol.* 12 517–533. 10.1038/nrm3151 21779028

[B23] Mines M. A. Goodwin J. S. Limbird L. E. Cui F.-F. Fan G.-H. (2009). Deubiquitination of CXCR4 by USP14 is critical for both CXCL12-induced CXCR4 degradation and chemotaxis but not ERK ativation. *J. Biol. Chem.* 284 5742–5752. 10.1074/jbc.M808507200 19106094 PMC2645827

[B24] Nagasawa T. Hirota S. Tachibana K. Takakura N. Nishikawa S. Kitamura Y. (1996). Defects of B-cell lymphopoiesis and bone-marrow myelopoiesis in mice lacking the CXC chemokine PBSF/SDF-1. *Nature* 382 635–638. 10.1038/382635a0 8757135

[B25] Nguyen D. H. Giri B. Collins G. Taub D. D. (2005). Dynamic reorganization of chemokine receptors, cholesterol, lipid rafts, and adhesion molecules to sites of CD4 engagement. *Exp. Cell Res.* 304 559–569. 10.1016/j.yexcr.2004.11.022 15748900

[B26] Pearson G. Robinson F. Beers Gibson T. Xu B. E. Karandikar M. Berman K. (2001). Mitogen-activated protein (MAP) kinase pathways: regulation and physiological functions. *Endocr. Rev.* 22 153–183. 10.1210/edrv.22.2.0428 11294822

[B27] Pinilla-Macua I. Watkins S. C. Sorkin A. (2016). Endocytosis separates EGF receptors from endogenous fluorescently labeled HRas and diminishes receptor signaling to MAP kinases in endosomes. *Proc. Natl. Acad. Sci. U.S.A.* 113 2122–2127. 10.1073/pnas.1520301113 26858456 PMC4776482

[B28] Ray P. Stacer A. C. Fenner J. Cavnar S. P. Meguiar K. Brown M. (2014). CXCL12-γ in primary tumors drives breast cancer metastasis. *Oncogene* 34 2043–2051. 10.1038/onc.2014.157 24909174 PMC4261050

[B29] Rosselli-Murai L. K. Yates J. A. Yoshida S. Bourg J. Ho K. K. Y. White M. (2018). Loss of PTEN promotes formation of signaling-capable clathrin-coated pits. *J. Cell Sci.* 131:jcs208926. 10.1242/jcs.208926 29588397 PMC5963840

[B30] Schmid S. L. (2017). Reciprocal regulation of signaling and endocytosis: implications for the evolving cancer cell. *J. Cell Biol.* 216 2623–2632. 10.1083/jcb.201705017 28674108 PMC5584184

[B31] Scita G. Di Fiore P. P. (2010). The endocytic matrix. *Nature* 463 464–473. 10.1038/nature08910 20110990

[B32] Sigismund S. Algisi V. Nappo G. Conte A. Pascolutti R. Cuomo A. (2013). Threshold-controlled ubiquitination of the EGFR directs receptor fate. *EMBO J.* 32 2140–2157. 10.1038/emboj.2013.149 23799367 PMC3730230

[B33] Sigismund S. Argenzio E. Tosoni D. Cavallaro E. Polo S. Di Fiore P. P. (2008). Clathrin-mediated internalization is essential for sustained EGFR signaling but dispensable for degradation. *Dev. Cell* 15 209–219. 10.1016/j.devcel.2008.06.012 18694561

[B34] Sigismund S. Woelk T. Puri C. Maspero E. Tacchetti C. Transidico P. (2005). Clathrin-independent endocytosis of ubiquitinated cargos. *Proc. Natl. Acad. Sci. U.S.A.* 102 2760–2765. 10.1073/pnas.0409817102 15701692 PMC549482

[B35] Sorkin A. von Zastrow M. (2009). Endocytosis and signalling: intertwining molecular networks. *Nat. Rev. Mol. Cell Biol.* 10 609–622. 10.1038/nrm2748 19696798 PMC2895425

[B36] Steel E. Murray V. L. Liu A. P. (2014). Multiplex detection of homo- and heterodimerization of g protein-coupled receptors by proximity biotinylation. *PLoS One* 9:e93646. 10.1371/journal.pone.0093646 24691126 PMC3972117

[B37] Stone M. B. Shelby S. A. Núñez M. F. Wisser K. Veatch S. L. (2017). Protein sorting by lipid phase-like domains supports emergent signaling function in B lymphocyte plasma membranes. *eLife* 6:e19891. 10.7554/eLife.19891 28145867 PMC5373823

[B38] Stone M. B. Veatch S. L. (2015). Steady-state cross-correlations for live two-colour super-resolution localization data sets. *Nat. Commun.* 6:7347. 10.1038/ncomms8347 26066572 PMC4467025

[B39] Tan X. Luo M. Liu A. P. (2018). Clathrin-mediated endocytosis regulates fMLP-mediated neutrophil polarization. *Heliyon* 4:e00819. 10.1016/j.heliyon.2018.e00819 30263974 PMC6157066

[B40] Wee P. Wang Z. (2018). Regulation of EGFR endocytosis by CBL during mitosis. *Cells* 7:257. 10.3390/cells7120257 30544639 PMC6315415

[B41] Yi T. Zhai B. Yu Y. Kiyotsugu Y. Raschle T. Etzkorn M. (2014). Quantitative phosphoproteomic analysis reveals system-wide signaling pathways downstream of SDF-1/CXCR4 in breast cancer stem cells. *Proc. Natl. Acad. Sci. U.S.A.* 111 E2182–E2190. 10.1073/pnas.1404943111 24782546 PMC4040611

[B42] Yoon S. Seger R. (2006). The extracellular signal-regulated kinase: multiple substrates regulate diverse cellular functions. *Growth Factors* 24 21–44. 10.1080/02699050500284218 16393692

[B43] Zou Y.-R. Kottmann A. H. Kuroda M. Taniuchi I. Littman D. R. (1998). Function of the chemokine receptor CXCR4 in haematopoiesis and in cerebellar development. *Nature* 393 595–599. 10.1038/31269 9634238

