# Supplementary material for: Clathrin Heavy Chain Knockdown Impacts CXCR4 Signaling and Post-translational Modification
Source: Front Cell Dev Biol. 2019 May 8;7:77. doi: 10.3389/fcell.2019.00077 (PMC6518350; doi:10.3389/fcell.2019.00077)
Supplement: Supplementary file 1 [file Image_1.pdf]

Clathrin heavy chain knockdown impacts CXCR4 signaling and post-translational modification

Maxwell S. DeNies [a], Luciana K. Rosselli-Murai [b], Santiago Schnell [a,c,d], Allen P. Liu [a,e,f,g *]

[a] Cellular and Molecular Biology Graduate Program, University of Michigan Medical School, Ann Arbor, Michigan, USA

[b] Department of Pharmacology, University of Michigan Medical School, Ann Arbor, Michigan, USA

[c] Department of Molecular & Integrative Physiology, University of Michigan Medical School, Ann Arbor, Michigan, USA

[d] Department of Computational Medicine & Bioinformatics, University of Michigan Medical School, Ann Arbor, Michigan, USA

[e] Department of Mechanical Engineering, University of Michigan, Ann Arbor, Michigan, USA

[f] Department of Biomedical Engineering, University of Michigan, Ann Arbor, Michigan, USA

[g] Department of Biophysics, University of Michigan, Ann Arbor, Michigan, USA

[*] Address correspondence to: Allen P. Liu (allenliu@umich.edu)

Supplemental Figures

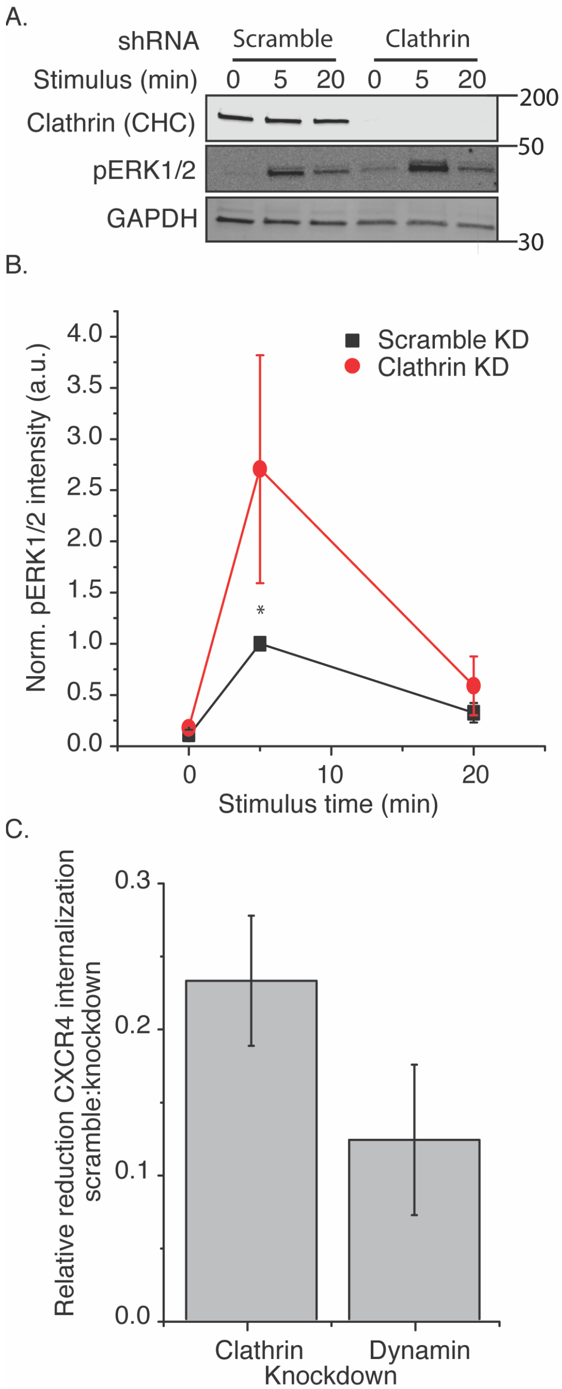

Supplemental Figure 1: *Clathrin knockdown reduces CXCR4 internalization and increases ERK1/2 phosphorylation in HeLa Cells.* **(A)** Representative western blot showing the effects of clathrin knockdown (shRNA 2755) in ERK1/2 phosphorylation in HeLa cells. **(B)** Quantification of relative CXCL12-induced ERK1/2 phosphorylation in HeLa cells. Relative ERK1/2 phosphorylation was normalized to ERK1/2 phosphorylated at 5 min post-CXCL12 stimulus of the scramble knockdown condition. **(C)** Relative reduction of CXCR4 internalization upon clathrin

knockdown (shRNA 2755 or 7981) by flow cytometry analysis (n = 3, mean +/- SEM, * denotes statistical significance $p < 0.05$).

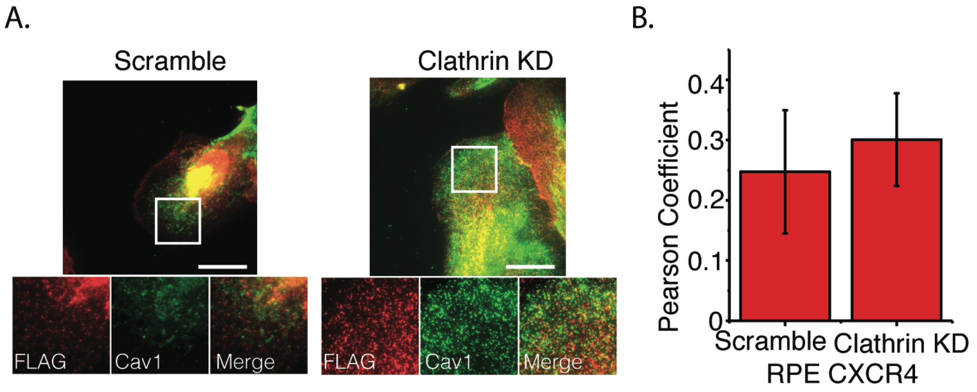

Supplemental Figure 2: *CXCR4 and caveolin-1 colocalization upon clathrin knockdown.* **(A)** Representative images of CXCR4 caveolin-1 colocalization in RPE cells stably overexpressing CXCR4 (scale bar 10 μm) +/- scramble or clathrin knockdown (shRNA 7981). Cells were serum-starved for 4 hrs and stimulated with 25 nM CXCL12 for 5 min. After immunofluorescence labeling (FLAG and caveolin-1 antibodies), cells were imaged by TIRF microscopy. **(B)** Quantification of CXCR4-caveolin-1 colocalization in cropped images (Pearson correlation coefficient, n = 6, mean +/- SEM) is plotted. While an increase in colocalization is observed with clathrin knockdown, it is not statistically significant.

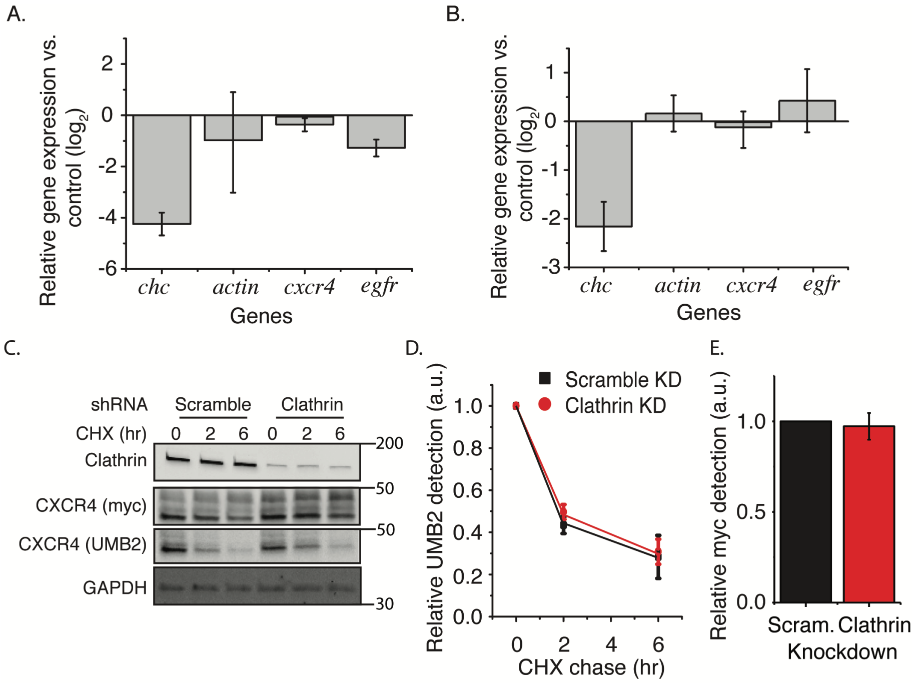

Supplemental Figure 3: *Clathrin knockdown does not change overexpressed CXCR4 degradation kinetics in RPE cells or mRNA levels in HeLa cells.* **(A-B)** RT-qPCR quantification of *clathrin heavy chain* (*chc*), *actin*, *cxcr4*, and *egfr* transcript levels upon clathrin knockdown (shRNA 2755) in **(A)** serum-free or **(B)** serum conditions. Relative transcript levels were calculated using the $\Delta\Delta C_t$ method and normalized to *gapdh.* The mean relative gene expression (log_2 transformed) +/- SD is plotted. Data was collected with duplicate technical replicates from 4 biologically independent experiments. **(C)** A representative western blot showing the effects of clathrin knockdown (shRNA 2755) on overexpressed CXCR4-myc protein degradation kinetics. In serum-supplemented media, cells were treated with 50 µM cycloheximide for the described time course and CXCR4 was detected using UMB2 and myc antibodies. **(D)** Relative UMB2 detection is plotted with mean +/- SEM from 3 independent experiments upon scramble or clathrin knockdown. Relative normalized CXCR4 detection was calculated by taking the ratio of GAPDH normalized UMB2 detection to the initial time point. No statistical significance was observed. Of note, CXCR4-myc constructs did not have as large of a decrease in UMB2 detection upon clathrin knockdown. This is potentially due to the fact that these experiments were conducted in serum or potential inhibition of receptor PTMs by myc addition to the receptor C-terminus. **(E)** Quantification of relative myc antibody detection (i.e. total CXCR4) upon scramble or clathrin knockdown (shRNA 2755). Total CXC4 protein levels were unchanged with clathrin knockdown. Data is plotted with mean +/- SEM from 3 independent experiments upon clathrin or scramble shRNA knockdown.

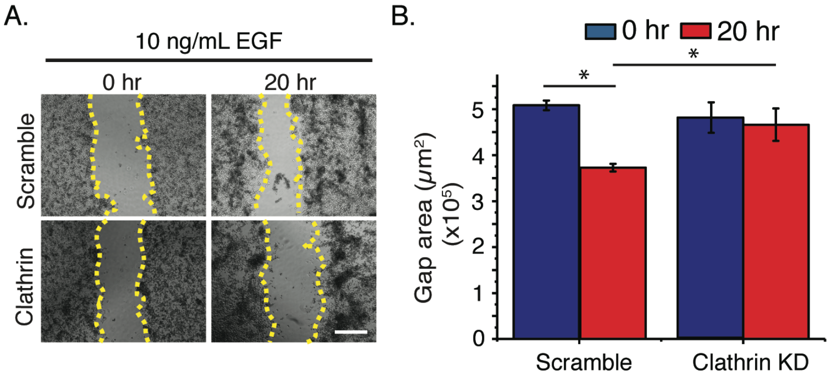

Supplemental Figure 4: *Clathrin knockdown decreases EGF-dependent HeLa cell migration.* **(A)** Representative images of a scratch assay time course. Five days post transduction with either scramble or clathrin (shRNA 2755 or 7981) knockdown HeLa cells were serum-starved for 4 hrs and treated with medium containing 10 ng/mL EGF in serum-free medium. Using a p200 pipet tip, a line was scratched on each plate and relative cell migration was measured by phase contrast microscopy at 0 hr or 20 hr post scratching. Cell boundaries used to calculate cell migration are outlined by the yellow dotted line. **(B)** Quantification of gap area (between dotted yellow boundaries) under each condition. Mean gap area +/- SEM is plotted from 3 independent experiments (* denotes statistical significance $p < 0.05$).

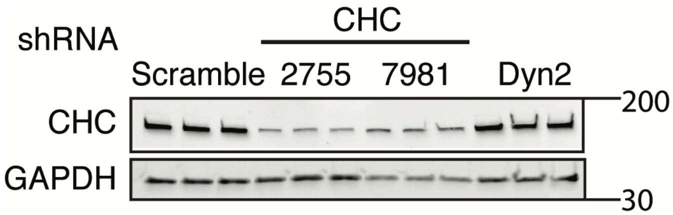

Supplemental Figure 5: *Clathrin heavy chain shRNA knockdown efficiency.* A representative western blot illustrating the relative knockdown efficiency of two independent clathrin shRNAs (2755 and 7981) compared to a scramble or dyanmin2 control shRNA (samples were loaded in triplicate).
